# Clinical characteristics and predictors of *Mycoplasma pneumoniae* pneumonia coinfection with *Bordetella pertussis* in children

**DOI:** 10.1097/MD.0000000000048911

**Published:** 2026-05-22

**Authors:** Chongyuan Lai, Shuping Peng, Xianfei Wu

**Affiliations:** a Department of Pediatrics, The First People’s Hospital of NanKang District, Ganzhou, Jiangxi, China; b Department of Pediatrics, The People’s Hospital of Baoan Shenzhen, Guangdong, China.

**Keywords:** *Bordetella pertussis*, children, etiology, *Mycoplasma pneumoniae*, pneumonia, predictor

## Abstract

This study aimed to investigate the clinical characteristics of *Mycoplasma pneumoniae* (MP) pneumonia coinfection with *Bordetella pertussis* (BP) in children and to determine the predictors of BP coinfection. Children with MP pneumonia (MPP) admitted to The First People’s Hospital of NanKang District between January 2024 and January 2025 were screened. Patients with BP coinfection were assigned to the study group, while those with mono-infection of MP were assigned to the control group at a 1:1 ratio, matched by age and admission time. Clinical data were compared between the 2 groups. Multivariate logistic regression analysis was performed to evaluate the association between BP coinfection, severe pneumonia, and azithromycin (AZM) treatment failure. Receiver operating characteristic (ROC) curve analysis was used to identify the potential predictive factors for BP coinfection. A total of 1475 hospitalized children with MPP were screened. Forty children were enrolled in the study and 40 in the control group. Children in the study group had a higher incidence of fever and spasmodic cough as well as a longer duration of fever and cough. They also exhibited an elevated white blood cell (WBC) count and lymphocyte (LYM) percentage, higher rates of severe pneumonia, oxygen therapy, bronchoalveolar lavage, methylprednisolone use, longer hospital stays, and higher hospitalization costs. Coinfection with BP was associated with severe MP pneumonia (*P* = .017) and treatment failure (*P* = .038). The area under the ROC curve (AUC) values for WBC count, LYM percentage, fever duration, and cough duration were 0.644 (95% CI: 0.521–0.768, *P* = .028), 0.641 (95% CI: 0.521–0.760, *P* = .032), 0.638 (95% CI: 0.516–0.761, *P* = .036), and 0.698 (95% CI: 0.576–0.820, *P* = .003), respectively. BP coinfection in children with hospitalized MPP is associated with an increased risk of severe pneumonia and treatment failure, which may complicate clinical management and increase disease burden. WBC count, LYM percentage, fever duration, and cough duration could serve as predictive factors for BP coinfection.

## 1. Introduction

Pertussis, an infectious disease caused by *Bordetella pertussis* (BP)^[1]^ and *Mycoplasma pneumoniae* pneumonia (MPP) induced by *Mycoplasma pneumoniae* (MP),^[2]^ is a common respiratory disorder in pediatric populations, with its prevalence showing an upward trend in recent years.^[3,4]^ Despite the high coverage of pertussis vaccination in China, the phenomenon of “pertussis resurgence.”^[5]^ Since the COVID-19 pandemic, MP has become one of the most frequently detected pathogens in pediatric community-acquired pneumonia (CAP).^[6]^ Clinical experience suggests that BP coinfection may exacerbate illness severity and complicate the management of MPP in children. However, studies specifically investigating BP coinfection in pediatric MPP remain limited, with most of the available evidence restricted to case reports.^[7]^ Thus, a comprehensive analysis of the clinical features of MPP patients with BP coinfection is urgently needed to fill this knowledge gap. Given the recent increase in the prevalence of both BP and MP, clarifying the impact of coinfection on the disease course and outcomes is of great clinical importance. This study aimed to investigate the clinical characteristics of pediatric MPP coinfection with BP and determine the clinical predictors of coinfection. We hypothesized that coinfection with BP is associated with increased disease severity and greater treatment challenges in pediatric MPP.

## 2. Materials and methods

### 2.1. Study children

This prospective study was conducted in the pediatric department of The First People’s Hospital of NanKang District (Jiangxi Province, China). The study period spanned from January 2024 to January 2025. The participants were 1475 children, ranging in age from 28 days to 14 years, who were hospitalized for MPP. Prior to the study initiation, approval was obtained from the Human Research Ethics Committee. All legal guardians of the enrolled children agreed to participate and signed the informed consent forms.

The inclusion criteria for the research subjects were as follows: Presence of respiratory symptoms such as fever, cough, asthma, dyspnea, expectoration; physical examination reveals decreased breath sounds or pulmonary rales; pneumonia confirmed by chest x-ray or computed tomography (CT) scan; laboratory confirmation of MP infection can be achieved through a positive MP-IgM serology test (enzyme immunoassay), a positive MP-DNA PCR test on a nasopharyngeal swab, or a positive MP antigen test; evidence of BP infection: a positive result from a nasopharyngeal swab BP-DNA PCR test.

Children were excluded from the study based on the following criteria: age < 1 month or >14 years; history of chronic respiratory disease; history of systemic diseases affecting the cardiovascular, hepatic, renal, hematological, neurological, or immunological system; and administration of systemic corticosteroids prior to hospital admission.

### 2.2. Pathogen detection

Respiratory pathogen detection was performed for all research subjects. MP infection was confirmed using the following methods: serologic testing using a Respiratory Antibody Quintuple Panel (including adenovirus [ADV], respiratory syncytial virus [RSV], MP, *Chlamydia pneumoniae* [CP], and Coxsackievirus, kit manufacturer: Beijing Innotech Biotechnology Co., Ltd., manual testing), pharyngeal swab analysis using a Respiratory Antigen Triple detection kit (including influenza A virus, influenza B virus, and MP, kit manufacturer: Beijing Innotech Biotechnology Co., Ltd., manual testing), serum MP-IgM detection (kit manufacturer: Beijing Innotech Biotechnology Co., Ltd., colloidal gold method), and pharyngeal swab MP-DNA detection (kit manufacturer: Daan Gene, testing instrument: Gentier 96R). BP infection was verified using pharyngeal swab BP-DNA detection (kit manufacturer: Daan Gene, testing instrument: Gentier 96R). Finally, screening for additional pathogens was performed using sputum culture and targeted next-generation sequencing (tNGS) with the Respiratory 100 panel (KingMed Diagnostics, Guangzhou, China), covering bacteria, mycobacteria, viruses, fungi, and specific drug-resistant sequences. To ensure accurate clinical interpretation, all tNGS results were independently reviewed by 2 experienced clinicians to distinguish between true infection and colonization.

### 2.3. Case definitions and classification

Children with MP infections were defined as those with laboratory-confirmed MP infections and negative test results for other respiratory pathogens. Children with coinfection were defined as those with laboratory-confirmed MP infection and concurrent BP detection. All participants were classified into a control group (children with mono-MP infections) and a study group (children with BP and MP coinfections) based on the etiological results.

### 2.4. Diagnostic criteria for severe pneumonia

All patients underwent chest computed tomography (CT) on admission. The severity of pneumonia, as assessed by CT scan or chest x-ray, was categorized as follows^[8]^ – Mild: less than one-third of the lung volume was affected. Moderate: one-third to one-half of the lung volume affected. Severe: more than half of the lung volume is affected.

In this study, the diagnostic criteria for severe pneumonia were based on Guidelines for the Management of Community-Acquired Pneumonia in Children (revised in 2023) issued by the Subspecialty Group of Respiratory Diseases of the Chinese Society of Pediatrics. The children with pneumonia were diagnosed with severe pneumonia. They met at least one of the following criteria: poor general condition; altered consciousness; and cyanosis, dyspnea, pulse oxygen saturation ≤92%, or a significant increase in breathing rate (≥70 breaths/min in infants and ≥50 breaths/min in older children). Ultra-high fever or persistent high fever lasting > 5 days; refusal of food or signs of dehydration; extensive pulmonary involvement (≥2/3 of a lobe or multilobar), pleural effusion, pneumothorax, atelectasis, pulmonary necrosis, or lung abscess; and extrapulmonary complications.

### 2.5. Treatment protocol

Upon admission, all children were administered azithromycin (AZM) as empirical treatment at a dose of 10 mg/kg/d for 5 days. If both clinical and radiographic improvements were observed, the treatment was continued for 2 or 3 courses. For children with severe or critical disease that would progress to refractory MPP (RMPP), intravenous methylprednisolone was administered to suppress the inflammatory response. The recommended dosage is 2 mg/kg/d, with a reduction in dosage after 3 days. This is in line with the current guideline recommendations.

Children who showed no improvement after the initial AZM course were assigned to the second-line therapy group. Escalation to second-line antibiotics was considered in cases of persistent fever (≥38.0°C for ≥72 hours) and/or radiographic progression (e.g., new or worsening pulmonary infiltrates) following the initiation of macrolide therapy. In accordance with national guidelines, second-line treatment regimens were as follows: for children aged ≥8 years, doxycycline 2 mg/kg every 12 hours for 10 days. Levofloxacin (8–10 mg/kg every 12 hours for 7–14 days) was administered to children aged 6 months to 5 years. For children aged 5 to 16 years: levofloxacin (8–10 mg/kg once daily for 7–14 days).

If children fail to recover after second-line treatment, the Chinese clinical guidelines for pertussis (revised in 2024) recommend trimethoprim-sulfamethoxazole (TMP-SMZ, at a dosage of TMP 4 mg/kg SMX 20 mg/kg every 12 hours for 10 days) as third-line treatment.

### 2.6. Assessment of therapeutic outcomes

Children were stratified based on their therapeutic response to the initial azithromycin (AZM) course as follows, with reference to published literature^[9]^ to minimize subjectivity – Good response: complete resolution of clinical symptoms related to pneumonia (including fever, cough, tachypnea, and chest distress) and/or significant improvement (≥50% reduction in lesion area) on chest imaging within 3 to 5 days of AZM initiation. Slow response: mild but measurable improvement in clinical symptoms (e.g., reduced frequency/duration of fever and cough) and chest imaging (20–49% reduction in lesion area) within 5 to 7 days of AZM initiation, without disease progression. No response: absence of any improvement or evident progression (e.g., increased lesion area on chest imaging and aggravated respiratory distress) in both clinical symptoms and chest imaging within 7 days of AZM initiation. All evaluations were performed independently by 2 attending pediatricians who were blinded to the group assignment, and any discrepancies were resolved by consensus to minimize subjective bias. Patients with a slow or no response were defined as having AZM treatment failure.

The utilization rates of oxygen therapy, mechanical ventilation, bronchoalveolar lavage (BAL), and second- and third-line treatment were calculated. Additionally, data related to disease burden in children, including hospital stays and hospital costs, were collected.

### 2.7. Data collection

The following data were collected from all enrolled participants – Demographics: age and sex. History: past medical history and vaccination status. Clinical symptoms: presence of fever, paroxysmal cough (which present with paroxysmal, violent coughing, frequently resulting in reddening of the face and often followed by a high-pitched inspiratory whoop sound, wheezing, dyspnea, along with the duration of fever and cough. A fever day was defined as any day on which the patient’s body temperature reached or exceeded 37.3°C at any point. Laboratory findings: MP drug-resistance genes: The rates of A2063G and/or A2064G point mutations in 23SrRNA; hematological parameters: white blood cell (WBC) count and the percentage of lymphocytes (LYM); inflammatory markers: C-reactive protein (CRP), procalcitonin (PCT), and interleukin-6 (IL-6); liver function: alanine aminotransferase (ALT); cardiac enzymes: creatine kinase(CK), lactate dehydrogenase (LDH), and hydroxybutyrate dehydrogenase (HBDH). Other markers: ferritin and d-dimer levels. Radiological findings: baseline chest CT manifestations.

### 2.8. Statistical analysis

Continuous variables with a normal distribution are presented as mean ± standard deviation (SD), whereas those with a non-normal distribution are reported as median and interquartile range (IQR). Categorical variables are presented as counts and percentages (n/%). Between-group comparisons were performed using the independent-samples *t* test for normally distributed continuous variables and Mann–Whitney *U* test for non-normally distributed variables. Categorical variables were compared using Pearson χ^2^ test or Fisher exact test when the expected frequency in any cell was <5. Multivariate logistic regression analyses were performed to assess the associations of BP coinfection with severe MPP and azithromycin treatment failure, and we restricted covariates to sex, age, and BP coinfection in each model to minimize overfitting and comply with the 10 events per variable guideline. Variables with statistically significant differences in univariate analysis were included in the receiver operating characteristic (ROC) curve analysis to evaluate their predictive value for BP coinfection. All statistical analyses were performed using the IBM SPSS Statistics for Windows (version 22.0; IBM Corp., Armonk). A 2-sided *P* < .05 was considered statistically significant.

## 3. Results

### 3.1. Children enrollment

A total of 1475 hospitalized children diagnosed with MPP were enrolled in this study. Of these, 106 children were diagnosed with BP pneumonia and 56 were confirmed to have BP–MP coinfection, yielding a coinfection rate of 3.79% in the overall MPP cohort. According on the exclusion criteria, 16 patients were excluded: 12 with coinfection by other respiratory pathogens, 3 with preexisting chronic respiratory diseases, and 1 who had received systemic corticosteroids prior to admission. Ultimately, 40 children with BP–MP coinfection were enrolled in the study group. Using 1:1 matching by age, sex, and time of admission, 40 patients with mono-MPP were included in the control group.

### 3.2. Demographic characteristics and clinical characteristics

All enrolled children routinely received the pertussis vaccine. The study group comprised 40 children, with no significant differences in age or sex compared with the control group. Seven (17.5%) patients were aged <2 years, 11 (27.5%) were 2 to 5 years old, 17 (42.5%) were 5 to 10 years old, and 5 (12.5%) were aged >10 years old in study group. Girls accounted for 57.5% and 45% of the study and control groups, respectively. Compared with the control group, the study group had a significantly higher incidence of fever (75% vs 32.5%, *P* < .001) and spasmodic cough (85% vs 10%, *P* < .001), as well as a longer duration of fever (median:5 days vs 4 days, *P* = .035) and cough (median: 10 days vs 6 days, *P* = .004). Detailed data are summarized in Table 1.

**Table 1:** Comparison of clinical date between 2 groups.

	Study group	Control group	*P* value
Demographic characteristics			
Girls, n (%)	23 (57.5%)	18 (45.0%)	.263^a^
Age (yr), median	5 (4–7)	6.5 (4–9)	.331^b^
Clinical characteristics			
Fever, n (%)	30 (75.0%)	13 (32.5%)	<.001^a^
Spasmodic cough, n (%)	34 (85%)	4 (10%)	<.001^a^
Asthma, n (%)	8 (20%)	4 (10%)	.210^a^
Dyspnea, n (%)	5 (12.5%)	2 (5%)	.432^a^
Fever duration (d), median	5 (4–7)	4 (3–5)	.035^b^
Cough duration (d), median	10 (6–15)	6 (4–7)	.004^b^
Laboratory finding			
MP drug resistance genes, n (%)	13 (32.5%)	11 (27.5%)	.626^a^
WBC (×10^9^), mean	10.28 ± 3.81	8.43 ± 2.81	.016^c^
LYM (%), mean	0.522 ± 0.177	0.613 ± 0.130	.011^c^
CRP (mg/L), median	5.32 (0.20–11.67)	5.83 (1.38–8.98)	.768^b^
PCT (ng/mL), median	0.037 (0.022–0.077)	0.036 (0.017–0.067)	.473^b^
IL-6 (pg/mL), median	11.1 (5.0–18.6)	9.1 (5.0–17.4)	.071^b^
LDH (U/L), mean	315.08 ± 64.80	303.13 ± 58.23	.557^c^
HBDH (U/L), mean	249.05 ± 55.40	239.33 ± 40.09	.371^c^
CK (U/L), median	117 (29.5–181)	96 (72–136.8)	.170^b^
ALT (U/L), median	14 (11–19)	13.5 (11.3–16.8)	.636
Ferritin (μg/L), median	119.5 (90.6–179.8)	113.5 (78.7–164.5)	.470^b^
IgE (IU/mL), median	101.6 (31.6–269.7)	96.5 (30.8–272.5)	.928^b^
Chest CT grading			.003^a^
Mild	16	14	
Moderate	19	8	
Severe	5	18	
Disease severity and treatment			
Severe pneumonia, n (%)	35 (87.5%)	25 (62.5%)	.010^a^
Oxygen therapy, n (%)	13 (32.5%)	4 (10%)	.014^a^
Mechanical ventilation, n (%)	0	0	–
BAL, n (%)	12 (30%)	3 (7.5%)	.010^a^
Methylprednisolone, n (%)	25 (62.5%)	14 (35%)	.014^a^
Second-line treatment, n (%)	14 (35%)	12 (25%)	.329^a^
Third-line treatment, n (%)	3 (7.5%)	0	.241^d^
Response to AZM, n			.025^d^
Good response	17	27	
Slow response	23	13	
No response	0	0	
Hospital stay (d), median	6 (5–8)	5 (5–6)	.002^b^
Hospital costs (yuan), mean	3151.98 ± 936.49	2627.75 ± 643.39	.005^c^

a Chi-square test.

b independent-samples Mann-Whitney U test.

c independent-samples t test.

d Fisher’s exact test.

ALT = alanine aminotransferase, AZM = azithromycin, BAL = bronchoalveolar lavage, CK = creatine kinase, CRP = C-reactive protein, CT = computed tomography, HBDH = hydroxybutyrate dehydrogenase, IL-6 = interleukin-6, LDH = lactate dehydrogenase, LYM = lymphocytes, MP = Mycoplasma pneumoniae, PCT = procalcitonin, WBC = white blood cell.

### 3.3. Comparisons of laboratory and radiographic findings

Compared with the controls group, the study group showed significantly elevated WBC count (10.28 × 10^9^/L vs 8.43 × 10^9^/L, *P* = .016) and percentage of LYM (20.5% vs 18.0%, *P* = .016), along with more severe chest CT grading (*P* = .003). No significant differences were found in the other laboratory parameters or radiographic findings (Table 1).

### 3.4. Comparisons of disease severity and treatment

The incidence of severe pneumonia was significantly higher in the study group than that in the control group (87.5% vs 62.5%, *P* = .010). The rates of oxygen therapy (32.5% vs 10%, *P* = .014), BAL (30% vs 7.5%, *P* = .010), and methylprednisolone use (62.5% vs 35%, *P* = .014) were all significantly higher in the study group. The response to AZM was poorer in the study group than in the control group (*P* = .025); however, the second- and third-line treatments did not differ significantly between the 2 groups (Table 1). Regarding disease burden, the hospital stay (median, 6 days vs 5 days, *P* = .002) and hospital costs (mean, 3151.98 yuan vs 2627.75 yuan, *P* = .005) were significantly higher in the study group (Table 1).

### 3.5. Coinfection was associated with severe pneumonia and treatment failure

To evaluate the association between BP coinfection, severe MPP, and azithromycin (AZM) treatment failure, 2 multivariate logistic regression models were constructed. After adjusting for sex and age, BP coinfection was significantly associated with severe MPP (odds ratio [OR], 4.042; 95% confidence interval [CI]: 1.280–12.680; *P* = .017; Table 2) and AZM treatment failure (OR, 2.649; 95% CI: 1.052–6.658; *P* = .038; Table 3).

**Table 2:** Multivariate regression analysis for severe MPP.

Variables	*P* value	OR	95% CI
Gender	.786	0.862	0.295–2.521
Age	.551	1.054	0.886–1.255
Coinfection	.017	4.042	1.280–12.680

CI = confidence interval, MPP = *Mycoplasma pneumoniae* pneumonia, OR = odds ratio.

**Table 3:** Multivariate regression analysis for treatment failure.

Variables	*P* value	OR	95% CI
Gender	.409	0.677	0.268–1.709
Age	.570	1.042	0.904–1.202
Coinfection	.038	2.649	1.052–6.658

CI = confidence interval, OR = odds ratio.

### 3.6. ROC analysis of the predictor of coinfection

**Figure 1.**
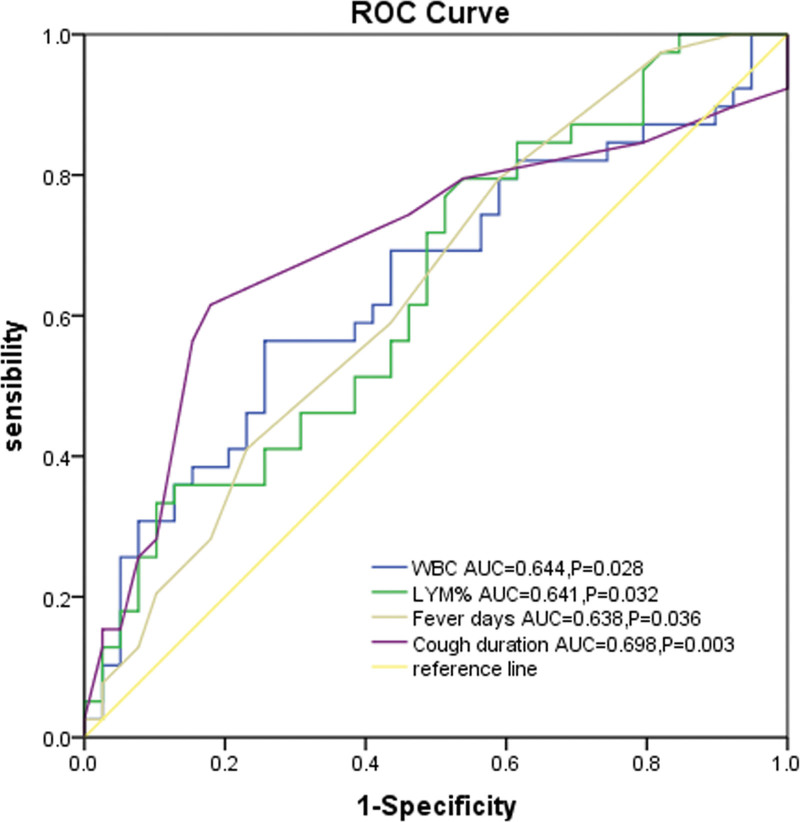
ROC analysis for the predictors of coinfection. AUC = area under the curve, LYM = lymphocytes, ROC = receiver operating characteristic, WBC = white blood cell.

ROC curve analysis was performed to evaluate the predictive value of indicators (identified from intergroup comparisons) for BP–MP coinfection. The area under the curve (AUC) for each indicator is shown in Figure 1. Of the laboratory findings, the AUC for WBC count and percentage of LYM was 0.644 (95% CI: 0.521–0.768, *P* = .028) and 0.641 (95% CI: 0.521–0.760, *P* = .032), with optimal cutoff value of 9.26 × 10^9^/L and 45.3%, respectively. The AUC for the duration of fever and cough were 0.638 (95% CI: 0.516–0.761, *P* = .036) and 0.698 (95% CI: 0.576–0.820, *P* = .003), respectively, with cutoff value of 3.5 days and 7.5 days (Fig. 1).

## 4. Discussion

In the present study, the BP coinfection rate among children with MPP was 3.79%. In terms of clinical characteristics, our study demonstrated that the study group (BP–MP coinfection) had a significantly higher incidence of spasmodic cough than the control group (85% vs 10%, *P* < .001). Spasmodic cough is a characteristic of pertussis-like syndrome^[10]^ and is frequently associated with pathogens including respiratory syncytial virus (RSV), adenovirus (ADV), BP, and MP. *Mycoplasma pneumoniae* infection is also a well-recognized etiological factor of pertussis-like syndrome,^[10]^ with a reported incidence of approximately 20%.^[11]^ These findings suggest that BP coinfection may increase the likelihood of pertussis-like syndrome in pediatric MPP. Additionally, our study showed a significantly higher fever rate (75% vs 32.5%, *P* < .001) and longer fever duration (5 days vs 4 days, *P* = .035) in the study group. Consistent with these results, a previous study^[9]^ reported that children with MPP and respiratory virus coinfection had a fever duration exceeding 6 days. Another study^[4]^ similarly demonstrated a higher proportion of fever in coinfected patients than in those with a single infection (59.8% vs 40.8%). These observations suggest that coinfection may impede pathogen clearance and amplify the host immune response, leading to prolonged inflammation. Furthermore, we found that the duration of cough was significantly longer in the study group (10 days vs 6 days, *P* = .004). MP and BP are common pathogens causing chronic cough.^[12]^ Since MP infection typically leads to a shorter cough duration than BP infection,^[11]^ our results suggest that BP coinfection may prolong the cough duration in children with MPP.

Regarding laboratory findings, we observed significantly higher white blood cell (WBC) counts (10.28 × 10^9^/L vs 8.43 × 10^9^/L, *P* = .016) and lymphocyte (LYM) percentages (0.48 vs 0.39, *P* = .016) in the study group. In 2022, Choo et al^[8]^ investigated the clinical characteristics of 145 children with MPP and reported significantly higher WBC counts in the respiratory virus coinfection group than the non-coinfection group (10.3 × 10^9^/L vs 8.5 × 10^9^/L, *P* = .026), however, no significant difference in LYM percentage was noted between the 2 groups. Notably, this study did not further analyze the specific pathogens involved in mixed infections. *Mycoplasma pneumoniae* pneumonia (MPP) infection stimulates lymphocyte proliferation, which is closely associated with the prognosis of MPP.^[13]^

In terms of radiologic findings, the chest CT grading was significantly more severe in the study group (*P* = .003). Consistently, our study demonstrated a significantly higher incidence of severe pneumonia in the study group (87.5% vs 62.5%, *P* = .010), and MPP coinfection was associated with severe MPP. These findings are consistent with those of a previous study involving 679 children with severe CAP,^[2]^ which found that sex, age, and bacterial coinfection are risk factors for severe MP-induced pneumonia. Consequently, we adjusted for sex and age when constructing the multivariate regression model. Regarding the mechanism by which coinfection aggravates MPP, animal studies^[14]^ have shown that coinfection can downregulate the expression of genes related to ciliary activity, alter multiple immune-related signaling pathways, and impair pulmonary defense mechanisms. These mechanistic findings derived from viral coinfection models may provide a potential interpretive framework for BP–MP coinfection in humans.

Although the prevalence of macrolide-resistant MP^[15]^ and BP^[16]^ has increased in Asia over the past decade, macrolides remain the first-line treatment for MPP^[17]^ and BP.^[16]^ Unfortunately, the study group showed a poorer response to AZM (*P* = .025). Multivariate analysis revealed that BP coinfection was associated with AZM treatment failure (OR, 2.649; 95% CI: 1.052–6.658; *P* = .038). This finding implies that BP coinfection may complicate the clinical management of pediatric MPP and contribute to the development of RMPP. These observations are consistent with those of previous studies on MPP coinfection with ADV.^[18,19]^ These results suggest that coinfections represent another significant etiology of RMPP.^[19]^ The rate of methylprednisolone use was significantly higher in the study group than in the control group (62.5% vs 35%, *P* = .014). Previous studies have suggested that corticosteroids may be beneficial in cases of RMPP,^[20]^ particularly when symptoms persist for more than a week despite macrolide therapy.^[21]^ Previous results have indicated that MPP coinfection with BP may exacerbate the disease course and contribute to severe pneumonia. Given their anti-inflammatory properties,^[9]^ corticosteroids are commonly used for the management of severe pneumonia. Therefore, corticosteroids may be considered as an adjunctive therapy for pediatric MPP coinfected with BP. Moreover, the rate of oxygen therapy was significantly higher in the study group than in the control group (32.5% vs 10%, *P *= .014). Pneumonia cases involving BP coinfection often require respiratory support.^[1]^ Accordingly, oxygen therapy was deemed necessary for children with MPP coinfected with BP. Additionally, this study found that the rate of BAL was significantly higher in the study group than in the control group (30% vs 7.5%, *P* = .010). BAL has been shown to reduce adverse pulmonary sequelae and improve clinical outcomes in children with MPP.^[22]^ Moreover, BAL can be performed in lobes exhibiting diffuse or localized infiltrates in infants with BP.^[17]^ Therefore, BAL may be considered for children with MPP coinfected with BP, when pulmonary infiltrates are present in the lung lobes. Finally, second-line antibiotics, such as doxycycline or levofloxacin, were administered to children with macrolide-resistant MPP, accounting for 15.12% of the cases.^[23]^ Once macrolide resistance was confirmed, second-line agents were promptly initiated to minimize the risk of complications.^[17]^ However, no significant difference in second-line treatment outcomes was observed between the 2 groups, which may be explained by the relatively small sample size of this study.

In terms of disease burden, hospital stay was longer in the study group (6 days vs 5 days, *P* = .002), which is consistent with a previous study^[24]^ on MPP coinfected with ADV in children (10 days vs 8 days, *P* = .001). Additionally, this study revealed significantly higher hospital costs in the study group (3151.98 yuan vs 2627.75 yuan, *P* = .005). Collectively, these results indicate a greater disease burden in pediatric MPP patients with BP coinfection. Therefore, enhancing the recognition of BP coinfection in children with MPP is crucial for reducing the associated disease burden. ROC curve analyses were performed to identify the potential predictors of BP coinfection with MPP. The AUC for WBC count was 0.644 (95% CI: 0.521–0.768, *P* = .028) at the optimal cutoff of 9.26 × 10^9^/L, and the AUC for lymphocyte percentage (LYM%) was 0.641 (95% CI: 0.521–0.760, *P* = .032) at 45.3%. For clinical symptoms, the AUC for fever duration was 0.638 (95% CI: 0.516–0.761, *P* = .036) with a cutoff of 3.5 days, and the AUC for cough duration was 0.698 (95% CI: 0.576–0.820, *P* = .003) with a cutoff of 7.5 days. Although these indicators showed statistically significant associations with BP coinfection, the overall AUC values indicated modest discriminative capacity. These predictors should be interpreted cautiously given their limited diagnostic performance and clinical utility. They may serve as supplementary clinical clues rather than independent diagnostic criteria for coinfection in pediatric MPP.

In conclusion, the coinfection rate of BP was 3.79% among children with MPP. This study confirmed the clinical significance of BP coinfection in children with MPP. Compared with single MPP, those coinfected with BP presented with a higher incidence of pertussis-like symptoms and experienced significantly longer durations of fever and cough. Moreover, BP coinfection is associated with more severe pneumonia and an increased risk of treatment failure in children with MPP. Adjunctive therapies including methylprednisolone, oxygen therapy, and BAL lavage were employed as part of the management strategy. Furthermore, we identified several independent risk factors for coinfection, including an elevated WBC count, percentage of lymphocytes, and prolonged duration of fever and cough. These findings offer a scientific basis for the early identification and management of BP coinfection in pediatric MPP. Nevertheless, this study has several limitations. This was a single-center investigation with a relatively modest sample size, which may have limited the statistical power and external generalizability of our findings. Consequently, the associated factors identified herein should be regarded as preliminary evidence rather than definitive predictive indicators, and further large-scale, multicenter prospective studies and biomedical regulation strategies^[25]^ are warranted to validate and extend our observations.

## Author contributions

**Conceptualization:** Chongyuan Lai.

**Data curation:** Chongyuan Lai.

**Formal analysis:** Shuping Peng.

**Funding acquisition:** Chongyuan Lai.

**Investigation:** Chongyuan Lai.

**Project administration:** Chongyuan Lai, Xianfei Wu.

**Writing – original draft:** Chongyuan Lai.

**Writing – review & editing:** Chongyuan Lai.
